# Systemic-to-pulmonary collateral flow associations with antegrade pulmonary flow in single ventricle patients: insights from cardiac magnetic resonance imaging

**DOI:** 10.3389/fcvm.2024.1304087

**Published:** 2024-02-22

**Authors:** Anna Yanovskiy, Laura Martelius, Nicolina Nyman, Teemu Vepsäläinen, Ilkka Mattila, Otto Rahkonen, Tiina Ojala

**Affiliations:** ^1^ HUS Medical Imaging Center, Helsinki University Hospital, University of Helsinki, Helsinki, Finland; ^2^ Department of Pediatric Cardiology, Children’s Hospital, Helsinki University Hospital and University of Helsinki, Helsinki, Finland; ^3^ Pediatric Cardiac Surgery, Children’s Hospital, Helsinki University and Helsinki University Hospital, Helsinki, Finland; ^4^ Department of Pediatric Cardiology, Children’s Hospital, HUS Medical Imaging Center, Helsinki University Hospital and University of Helsinki, Helsinki, Finland

**Keywords:** single ventricle, systemic to pulmonary collateral flow, pulmonary flow, cardiac magnetic resonance, PVRI

## Abstract

**Purpose:**

In the palliated single ventricle anomalies, a considerable amount of the aortic flow may be absorbed by the systemic-pulmonary collateral flow (SPCF), which can be noninvasively assessed by cardiac magnetic resonance (CMR). The aims of this study were to (1) identify factors associated with SCPF in pediatric single ventricle patients, and (2) establish a cutoff values indicating an association between SCPF and a reduction in antegrade pulmonary flow.

**Methods:**

A retrospective single-tertiary-center cohort study included 158 consecutive CMR studies of patients with a single ventricle. In the uni- and multivariable analysis, SPCF was presented as a percentage of the total pulmonary venous flow (SPCF_%PV_). The minimal clinically important difference in Q_P_/Q_S_ ratios was estimated as ≥0.50, and an optimal cutoff value was defined using the receiver operating characteristic (ROC) curve.

**Results:**

SPCF_%PV_ was significantly smaller in the post-total cavopulmonary connection (TCPC) group than in the pre-TCPC patients (*p* < 0.001). The patient's higher age and a higher antegrade pulmonary flow were associated with a lower SPCF_%PV_. A negative weak association was observed between the SPCF_%PV_ and systemic saturation (*r* = −0.39, *p* < 0.001). SPCF_%PV_ did not associate with ventricular volumes nor ejection fraction. The SPCF_%PV_ was significantly smaller in patients that were palliated primarily with a pulmonary artery banding compared to those palliated with a BT-shunt (*p* = 0.002) or RV-PA- shunt (*p* = 0.044). In the ROC analysis, for pre-TCPC patient's, a cutoff of SPCF_%PV_ 42% yielded a sensitivity of 100% and specificity of 80% for significantly reduced antegrade pulmonary flow (AUC 0.97). In the post-TCPC group, the optimal SPCF_%PV_ cutoff was 34% (sensitivity 100%, specificity 98%, AUC 0.99).

**Conclusion:**

SPCF results in a considerable left-to-right shunt, which subsequently diminishes spontaneously after TCPC. Our findings indicated that for pre-TCPC patients, an SPCF%PV threshold of 42% (sensitivity 100%, specificity 80%), and for the post-TCPC group, a threshold of 34% (sensitivity 100%, specificity 98%) were effective in identifying reduced antegrade pulmonary flow.

## Introduction

Patients with a single ventricle have a complicated physiology requiring staged palliative correction in early childhood. During the bidirectional Glenn (BDG) and total cavopulmonary connection (TCPC) procedures, the pulmonary arteries are directly linked to the superior and inferior vena cavae, respectively, causing antegrade flow to the lungs with no pulsatility and reduced velocity. Additionally, single ventricle patients develop systemic to pulmonary collateral flow (SCPF) which may absorb a substantial amount of the aortic flow and reduce the effective cardiac index (1). The impact of SCPF on the pulmonary antegrade flow is still a matter of debate (2). SCPF is more prominent in the pre-TCPC stage than the post-TCPC stage. Previous studies (3–6) suggest that SCPF results from insufficiency in the antegrade pulmonary blood flow, deterioration of the systemic oxygen saturations, insufficiency in the vascular bed, and high pulmonary vascular resistance index (PVRi). High SCPF is recognized as a source of error in the invasive assessment of PVRi in single ventricle patients, as it assumes a single source of blood flow (7).

Cardiac magnetic resonance (CMR), using a 2-dimensional phase-contrast, allows for a noninvasive, accurate quantification of the amount of SCPF. Therefore, CMR is increasingly used in clinical evaluation of single ventricle patients, giving information on changes in the amount of SCPF during the follow-up. The aims of this study were to (1) identify factors associated with SCPF in pediatric single ventricle patients, and (2) assign cutoff values, where SCPF associates with decreased antegrade pulmonary flow.

## Materials & methods

### Patient selection

This retrospective single-tertiary-center cohort study provides data from 158 consecutive CMR studies of patients with single ventricle anomalies (Table 1). All congenital heart surgeries and single ventricle CMR examinations in Finland are centralized in one tertiary center, the pediatric cardiac unit at Helsinki University Hospital. The CMR examination was performed as a part of the clinical follow-up between January 2017 and June 2021. Hemodynamic data derived from CMR were retrospectively gathered from clinical reports. However, during the primary CMR analysis, each patient underwent dual analyses by the same experienced cardiologist (TO) and radiologist (LM) pair, specialized in single ventricle CMR examinations. Additionally, these patients acted as their own controls due to the multiple flow measurements conducted, offering internal validation within each individual. This dual control system reduces the risk for significant measurement errors. The clinical data, including the diagnosis, operations, interventions, and data regarding the AV-valve function were collected from the clinical and echocardiographic records.

**Table 1:** Demographic and clinical parameters, *n* = 158.

	pre-TCPC, (*n* = 27, 17%)	post-TCPC, (*n* = 131, 83%)	*p*-value
Male	14 (52%)	80 (61%)	0.374
Age at examination, years	2.7 (2.5; 3.1)	14.5 (10.6; 16.4)	<0.001
Weight at examination, kg	13 (11, 15)	50 (35, 62)	<0.001
Diagnosis, *n* (%)			
HLHS	9 (33%)	65 (50%)	0.123*
Non-HLHS	18 (67%)	66 (50%)	
Type of functional single ventricle, *n* (%)			
Right (RV)	10 (37%)	83 (63%)	0.027*
Left (LV)	14 (52%)	43 (33%)	
RV + LV	3 (11%)	5 (4%)	
Source of pulmonary blood flow during pre-Glenn phase, *n* (%)			
Blalock–Taussig	13 (48%)	51 (39%)	0.039*
Right ventricle-to-pulmonary artery	11 (41%)	30 (23%)	
Pulmonary artery banding	2 (7%)	21 (16%)	
No need for shunt or band/data not available	1 (4%)	29 (22%)	
Atrioventricular valve regurgitation, *n* (%)			
No regurgitation	10 (37%)	28 (21%)	0.228*
Mild (<10%)	14 (52%)	94 (72%)	
Moderate (11%–30%)	2 (7%)	3 (2%)	
Significant >30%)	0	1 (1%)	
No data available	1 (4%)	5 (4%)	
Status of fenestration at examination, *n* (%)			
Non-fenestrated	NA	14 (11%)	NA
Spontaneously closed		56 (43%)	
Transcatheter closure		36 (27%)	
Open fenestration		11 (8%)	
Fenestration without detectable flow		12 (9%)	
Reoperated TCPC		2 (2%)	
Follow-up after TCPC, years	NA	11.5 (7.3; 13.5)	NA
PVP, mmHg	13 (11; 15)	15 (13;16)	0.003

Data presented as median (25th and 75th percentile), or number (percentage). HLHS, hypoplastic left heart syndrome; PVP, peripheral venous pressure; TCPC, total cavopulmonary connection.

* Groups comparisons.

### Imaging

CMR was performed with a 1.5 T scanner (Philips Achieva or Ingenia), using a gadoteric acid (279.3 mg/ml, 0.2 ml/kg) contrast. Supplementary Table S1 summarizes the CMR parameters. A coronal fat-saturated single-shot T2-weighted non-contrasted lymphangiography was included in the CMR evaluation to detect neck lymphatic collaterals. Neck and thorax lymphatic collaterals were classified according to Biko et al. (8).

The left cubital vein was used for intravenous access. Additionally, the peripheral venous pressure (PVP) was measured from this cannula. The systemic oxygen saturation was immediately measured (Philips IntelliVue MX450) beforehand, and in the case of anesthesia study, during the examination.

Flow-measurements were performed in the following vascular structures: ascending aorta (AAo), descending aorta (DA), superior vena cava (SVC)/Glenn shunt, inferior vena cava (IVC), TCPC tunnel (in post-TCPC patients), right pulmonary artery (RPA), left pulmonary artery (LPA), right pulmonary veins (RPV), and left pulmonary veins (LPV). Ventricular end-diastolic volumes (EDV), end-systolic volumes (ESV), and ejection fraction were measured from the ventricular short-axis cine-images (9). Table 2 describes equations for the calculation of hemodynamic parameters. The McGoon ratio was calculated by the summation of right and left pulmonary artery diameter, divided by the diameter of descending aorta at the level of diaphragm.

**Table 2:** Methods and equations used for calculation of hemodynamic parameters.

Parameter	Equation
Effective cardiac index (CI = Q_s_)	Q_SVC _+ Q_DA_
Antegrade pulmonary flow (Qp_PA)_	Q_RPA_ + Q_LPA_
Total pulmonary flow (Qp_PV_)	Q_RPV _+ Q_LPV_
Systemic to pulmonary collateral flow (SPCF)	Qp_PV_ – Qp_PA_
Percentage of collateral flow (SPCF_%PV_)	SPCF/Qp_PV_
Modified McGoon ratio	LPA (mm) + RPA (mm)/DA (mm)
Q_P_/Q_S_ ratio is calculated in two ways	Qp_PA_/Q_S_ and Qp_PV_/Q_S_
Delta Q_P_/Q_S_	(Qp_PV_/Q_S_) – (Qp_PA_/_S_)

McGoon ratio was calculated by measuring the smallest hilar pulmonary arteries diameters and the diameter of the ascending aorta at the level of the diaphragm. DA, descending aorta; LPA, left pulmonary artery; LPV, left pulmonary vein; Q_DA_, blood flow in descending aorta; Q_LPA_, blood flow in left pulmonary artery; Q_LPV_, blood flow in left pulmonary vein; Q_RPA_, blood flow in right pulmonary artery; Q_RPV_, blood flow in right pulmonary vein; Q_s_, systemic flow; Q_SVC_, blood flow in superior vena cava; RPA, right pulmonary artery; RPV, right pulmonary vein.

In the cohort of 19 patients who underwent catheterization after the CMR study PVRi was assessed through three distinct methodologies: (1) utilizing catheterization data alone, (2) employing hybrid data that integrates catheterization data with CMR-derived antegrade pulmonary flow (Qp_PA_), and (3) CMR-derived pulmonary venous flow (Qp_PV_).

### Statistical analysis

All statistical calculations were performed using “IBM SPSS Statistic v.27”. Variables were tested for normality and presented as the median (25th and 75th percentile) and mean (standard deviation SD) as appropriate.

The statistical significance regarding the differences in demographic, clinical, and CMR-derived parameters between the pre-TCPC and post-TCPC groups was computed, using either the Mann–Whitney, two-sample *t*-test, or Chi square test. The paired samples t-test was used for the comparison of the pulmonary flow in pulmonary veins and arteries.

In the uni- and multivariable analysis for combined groups, SPCF was presented as a percentage of the pulmonary flow (SPCF_%PV_). *Univariable analysis for SPCF_%PV_*: The differences in variables with two categories were examined using the Mann–Whitney *U*-test, and the differences in variables with more than two categories using the Kruskal–Wallis test with pairwise comparisons by a Dunn-Bonferroni correction. *The association* between continuous variables and SPCF_%PV_ was calculated using Spearman's correlation. *Multiple risk factor analysis for SPCF_%PV_*: Factors significantly associated with SPCF_%PV_ in univariable models were included in a multivariable linear model (backward method). SPCF_%PV_ values were naturally log-transformed for the multivariable linear model due to a positively skewed distribution.

Association between the PVP and grade of the lymphatic collaterals was computed using a one-way ANOVA and Tukey *post hoc* test.

Differences in the McGoon ratio in patients with different types of pre-Glenn shunts were tested using a Kruskal–Wallis test with pairwise comparisons by a Dunn–Bonferroni correction. A *p*-value less than 0.05 was considered significant.

Receiver operating characteristic curve (ROC) and cutoff analysis: The minimal clinically important difference (MCID) in Qp/Q_S_ ratios was estimated as ≥0.50. An optimal cutoff value for SPCF_%PV_ was defined using the Youden index and the nearest-upper-left-corner approach.

Bland-Altman analysis was used for assessing agreement between catheterization and hybrid CMR PVRi estimates.

### Ethics

The study was approved by the Ethics Committee of the Helsinki University Hospital (HUS/108/2017, study permission HUS/151/2022).

## Results

All 158 univentricular patients underwent a bidirectional Glenn operation, 131 (83%) patients completed a TCPC operation, and 27 (17%) patients were at the pre-TCPC stage. The demographic data is presented in Table 1. None of the patients had a history of catheter embolization in the aorta-pulmonary collaterals.

In our cohort, the pulmonary venous flow [mean 3.2 (0.8) L/min/m^2^] was significantly higher than the flow in pulmonary arteries [mean 2.6 (0.9) L/min/m^2^], respectively; *p* < 0.001. The median SPCF was 15% (9%, 25%) of the total pulmonary blood flow. SPCF_%PV_ was significantly smaller in the post-TCPC group [median 12% (9%, 20%)] compared to the pre-TCPC group [median 41% (25%, 46%)], (respectively; *p* < 0.001) (Table 3, Supplementary Figures S1, S2).

**Table 3:** CMR derived anatomical and hemodynamic parameters.

	pre-TCPC, *n* = 27	post-TCPC, *n* = 131	*p*-value
EDV, ml/m^2^	99 (76; 107)	80 (69; 94)	0.006
ESV, ml/m^2^	37 (28; 50)	34 (26; 46)	0.441
EF, %	59 (9)	56 (9)	0.06
SV, ml/m^2^	48 (43; 59)	44 (37;50)	0.009
Descending aorta, diameter, mm	8 (8; 10)	13 (12; 15)	<0.001
RPA, diameter, mm	8 (2)	13 (4)	<0.001
LPA, diameter, mm	7 (2)	11 (3)	<0.001
McGoon ratio	1.7 (0.5)	1.8 (0.5)	0.266
Ascending aorta flow, L/min/m^2^	4.2 (3.6; 4.7)	3.2 (3.0; 3.9)	<0.001
Descending aorta flow, L/min/m^2^	1.6 (0.3)	1.9 (0.5)	<0.001
SVC (Glenn) flow, L/min/m^2^	1.5 (1.1; 1.8)	1.0 (0.8; 1.2)	<0.001
QpPA, L/min/m^2^	1.6 (0.6)	2.8 (0.8)	<0.001
QpPV, L/min/m^2^	2.5 (2.2; 3.2)	3.2 (2.9; 3.8)	<0.001
Qs, L/min/m^2^	2.8 (1.0)	2.9 (0.6)	0.679
Qp_PA_/Qs	0.6 (0.3)	1.0 (0.1)	<0.001
Qp_PV_/Qs	0.9 (0.8; 1.2)	1.1 (1.1; 1.2)	0.002
Delta Qp/Qs	0.4 (0.2; 0.5)	0.1 (0.1; 0.2)	<0.001
SPCF, L/min/m^2^	1.0 (0.6; 1.4)	0.4 (0.3; 0.6)	<0.001
SPCF_%PV_	41% (25%; 46%)	12% (9%; 20%)	<0.001
Lymphatic neck and thorax collaterals:	*n* = 22/27 (81%)	*n* = 109/131 (83%)	0.044*
No visible lymphatic collaterals	15 (68%)	42 (39%)	
Type 1	3 (14%)	37 (34%)	
Type 2	2 (9%)	24 (22%)	
Type 3	2 (9%)	6 (5%)	
Type 4	0	0	

Data presented as median (25th and 75th percentile), mean (SD) or number (percentage). EDV, end diastolic volume; ESV, end systolic volume; EF, ejection fraction; SV, stroke volume; CI, cardiac index; LPA, left pulmonary artery; RPA, right pulmonary artery; SPCF, systemic-to-pulmonary collateral flow.

* Groups comparisons.

### Univariate analysis for systemic-to-pulmonary collaterals

The results of the univariate analysis are presented in Table 4. SPCF_%PV_ was weakly negatively associated with the patient's age and moderately with the weight (*r* = −0.39, *r* = −0.46 respectively, *p* < 0.001), but it was not associated with the duration of time within the Fontan circulation (*r* = −0.099. *p* = 0.259). A weak negative association was observed between the SPCF_%PV_ and systemic saturation (*r* = −0.39, *p* < 0.001), and the LPA and RPA diameters (*r* = −0.33, *p* < 0.001; *r* = −0.41, *p* < 0.001, respectively), as well between SPCF_%PV_ and both pulmonary arterial and venous blood flow (*r* = −0.58, *p* < 0.001; *r* = −0.16, *p* = 0.044, respectively). SPCF%_PV_ was smaller in patients with a pulmonary artery banding in comparison to Blalock–Taussig (BT) or to the right ventricle-to-pulmonary artery (RV-PA) shunt (*p* = 0.035).

**Table 4:** Univariate analysis of SPCF%_PV_.

Variable	SPCF%_PV_	Correlation coefficient	*p*-value
Age, years		−0.39	<0.001
Weight, kg		−0.46	<0.001
Gender			
Male	13 (9, 25)		0.176
Female	16 (10, 25)		
Type of single ventricle			
HLHS	16 (10, 26)		0.190
Non-HLHS	13 (9, 24)		
Atrioventricular valve regurgitation			
Yes	14 (9, 25)		0.354
No	16 (11, 24)		
Status of fenestration by the time of CMR imaging			
Non-fenestrated	11 (8, 16)		0.054*
Closed (spontaneously, transcatheter closure or no detectable flow)	12 (9, 19)		
Open fenestration	36 (9, 46)		
Functional single ventricle:			
RV	15 (10, 24)		0.908*
LV	14 (9, 26)		
RV + LV	14 (7, 36)		
Follow-up after TCPC, years		−0.099	0.259
Source of pulmonary blood flow during pre-Glenn phase			
Blalock–Taussig	16 (10, 35)		0.035*
Right ventricle-to-pulmonary artery	16 (10, 32)		
Pulmonary artery banding	10 (7, 17)		
Saturation at CMR		−0.39	<0.001
Diameter of descending aorta, mm		−0.36	<0.001
LPA, mm		−0.33	<0.001
RPA, mm		−0.41	<0.001
QpPA, L/min/m^2^		−0.58	<0.001
QpPV, L/min/m^2^		−0.16	0.044
Qs flow (effective CI), L/min/m^2^		−0.29	<0.001
Delta Qp/Qs		0.97	<0.001
McGoon		−0.13	0.122
ESV, ml/m^2^		0.07	0.387
EVD, ml/m^2^		0.11	0.149
EF, %		0.001	0.994
PVP, mmHg		−0.05	0.551
Lymphatic neck and thorax collaterals:			
No visible lymphatic collaterals	15 (10; 34)		0.491*
Type 1	16 (10; 25)		
Type 2	14 (9; 19)		
Type 3	14 (11; 36)		

Data presented as median (25th and 75th percentile).

* Groups comparisons.

SPCF_%PV_ was not associated with gender, the diagnosis of HLHS, the absence or presence of atrioventricular valve regurgitation, the status of fenestration by the time of CMR imaging, or a type of functional single ventricle. SPCF_%PV_ did not correlate with the ventricular volumes, ejection fraction, or PVP.

Neck and thorax lymphatic collaterals assessments were available for 131 (83%) patients. The most severe type of lymphatic collaterals (type 4) was not observed in this cohort. There were more lymphatic abnormalities in post-TCPC group 67/109 (61%) than in the pre-TCPC group 7/22 (32%), (*p* = 0.039), but no difference was found in more severe (type 3) thorax lymphatic collaterals between the groups (*p* = 0.798). SPCF_%PV_ was not associated with the presence of neck or thorax lymphatic abnormalities (*p* = 0.315), nor with their grade (*p* = 0.491).

### Multiple risk factor analysis for systemic-to-pulmonary collaterals

Parameters that showed a significant association with SPCF_%PV_ in the univariate analysis were included in the multivariable model (Table 5). The patient's higher age and higher antegrade pulmonary flow Qp_PA_ were associated with a lower SPCF_%PV_. The SPCF_%PV_ was significantly smaller in patients that were palliated primarily with a pulmonary artery banding compared to those palliated with a BT-shunt (*p* = 0.002) or RV-PA- shunt (*p* = 0.044). Patients with a pulmonary artery banding had a significantly higher McGoon ratio compared to the BT-shunt (*p* = 0.007) and RV-PA-shunt (*p* = 0.001).

**Table 5:** Multivariable linear model (*n* = 128).

Variable	Adjusted *β* (SE)^a^	*p*-value
Age at CMR^b^	−0.044 (0.013)	<0.001
Pulmonary flow Qp_PA_, L/min/m^2†^	−0.324 (0.075)	<0.001
Source of pulmonary blood flow during pre-Glenn phase		
Blalock–Taussig	2.908 (0.082)	0.003*
Pulmonary artery banding	2.360 (0.136)	
Right ventricle-to-pulmonary artery	2.775 (0.103)	

β, regression coefficient for one-unit increase in continuous factors and adjusted mean for types of Norwood shunt; SE, standard error; Qp_PA_, antegrade pulmonary flow.

^a^ Adjusted for other variables included in the model, log-transformed values were used in the analysis.

^b^ Continuous factor.

* Significant difference in pairwise comparisons in patients with a pulmonary artery banding compared to Blalock–Taussig (*p* = 0.002) and right ventricle-to-pulmonary artery (*p* = 0.044) using Tukey's adjustment.

### Analysis of the cutoff for SPCF_%Pv_

We calculated SPCF_%PV_ cutoffs for the cohort overall and separately for subgroups.

Based on the findings of the ROC analysis (AUC = 0.97) for the entire cohort, the most relevant change in Qp/Qs suggested an optimal cutoff of SPCF_%PV_ 34% with a sensitivity of 100% and specificity of 91%. Cutoff values for SPCF_%PV_ between 24% and 34% showed a sensitivity of 100% and specificity over 80% (Supplementary Table S2).

In subgroup analysis, for pre-TCPC patient's, cutoff of SPCF_%PV_ was 42% (sensitivity 100%, specificity 80%, AUC 0.97) (Supplementary Table S3). In the post-TCPC group, the optimal SPCF_%PV_ cutoff was 34% (sensitivity 100%, specificity 98%, AUC 0.99) (Supplementary Table S4).

### Comparison of PVRi estimates

A diagnostic and/or interventional cardiac catheterization was conducted for 19 patients following CMR, with a mean time interval of 63 days (standard deviation 67 days, ranging from 0 to 217 days). In 17/19 patients (5 pre-TCPC and 12 post-TCPC) we were able to calculate PVRi based on both data: catheterization and CMR. In 6/17 patients SPCF_%PV_ exceeded cutoffs (pre-TCPC SPCF_%PV_ >42%; post-TCPC SPCF_%PV_ >34%) (Table 6).

**Table 6:** Pulmonary vascular resistance index (PVRi) calculations represented for patients below and above SPCF_%PV_ cutoff (pre-TCPC SPCF_%PV_ 42%; post-TCPC SPCF_%PV_ 34%).

Patients with SPCF_%PV_ below cutoffs	SPCF_%PV_ (%)	PVRi cath (WU.m^2^)	PVRi CMR QpPA (WU.m^2^)	PVRi CMR QpPV (WU.m^2^)
1.	0	1.6	1.5	1.5
2.	3	2.6	2.1	2.1
3.	3	2.1	1.7	1.6
4.	6	1.0	0.9	0.8
5.	7	1.3	1.5	1.4
6.	14	2.2	2.0	1.7
7.	15	1.9	3.5	3.0
8.	16	2.8	1.9	1.6
9.	19	1.9	2.9	2.4
10.	25	1.5	1.7	1.3
11.	27	2.1	1.9	1.4
Summary (median, 25th and 75th percentile)	14 (3; 19)	1.9 (1.5; 2.2)	1.9 (1.5; 2.1)	1.6 (1.4; 2.1)
Patients with SPCF_%PV_ above cutoffs	SPCF_%PV_ (%)	PVRi cath (WU.m^2^)	PVRi CMR QpPA (WU.m^2^)	PVRi CMR QpPV (WU.m^2^)
12.	43	2.2	2.5	1.4
13.	44	2.2	6.0	3.3
14.	46	2.5	3.3	1.8
15.	46	0.9	1.3	0.7
16.	55	2.8	5.6	2.5
17.	88	2.5	12.0	1.5
Summary (median, 25th and 75th percentile)	46 (44; 63)	2.4 (1.9; 2.6)	4.5 (2.2; 7.5)	1.7 (1.2; 2.7)

CMR QpPA, CMR antegrade pulmonary flow; CMR QpPV, CMR pulmonary venous flow; SPCF, systemic-to-pulmonary collateral flow.

The agreement between techniques was assessed using Bland-Altman analysis, and the results are displayed in Table 7. Notably, when hybrid data incorporating antegrade pulmonary flow were used, especially in cases where SPCF%_PV_ exceeded 34%, we observed wide limits of agreement, indicating substantial clinical discrepancies. On the other hand, when comparing PVRi with hybrid data utilizing pulmonary venous flow, we found an acceptable level of agreement, as illustrated in Table 7.

**Table 7:** Differences in pulmonary vascular resistance index (PVRi, WU.m^2^) measurements using cardiac magnetic resonance (CMR) and catheterization (cath) fick method.

	Mean difference	95% CI of mean difference	Limits of agreement
Comparison of PVRi, all available PVRi calculations, *n* = 17			
PVRi CMR QpPA – PVRi cath	1.1	−0.2; 2.4	−3.8; 5.9
PVRi CMR QpPV – PVRi cath	−0.2	−0.6; 0.1	−1.5; 1.0
Comparison of PVRi in patients with SPCF_%PV_ below cutoffs^a^, *n* = 11/17			
PVRi CMR QpPA – PVRi cath	0.1	−0.4; 0.5	−1.4; 1.5
PVRi CMR QpPV – PVRi cath	−0.2	−0.6; 0.2	−1.4; 1.0
Comparison of PVRi in patients with SPCF_%PV_ above cutoffs, *n* = 6/17			
PVRi CMR QpPA – PVRi cath	2.9	−0.7; 6.6	−3.9; 9.8
PVRi CMR QpPV – PVRi cath	−0.3	−1.1; 0.5	−1.8; 1.2

CMR QpPA – CMR antegrade pulmonary flow, CMR QpPV – CMR pulmonary venous flow, SPCF – systemic-to-pulmonary collateral flow.

^a^ SPCF_%PV_ cutoff for pre-TCPC patients 42% and post-TCPC 34%.

## Discussion

In this study, we evaluated SPCF and its association with the antegrade pulmonary flow in single ventricle patients during pre- and post-TCPC stages. This is the first investigation to establish the cutoff value for SPCF%_PV_ that indicates a noteworthy disparity in antegrade pulmonary flow. Our findings revealed, for pre-TCPC patients, an SPCF%_PV_ threshold of 42% (sensitivity 100%, specificity 80%), and for the post-TCPC group, a threshold of 34% (sensitivity 100%, specificity 98%) in identifying reduced antegrade pulmonary flow.

As shown previously (10, 11–13), SPCF was significantly smaller in patients after the TCPC than in the patients at the pre-TCPC stage. In contrast to some studies (3, 11), no association was revealed with follow-up time after the TCPC and SPCF. The decrease in SPCF is likely to occur shortly after surgery, presumably before the CMR assessment conducted in our study. In the multivariate analysis, associated factors for SPCF_%PV_ were the patient's age, antegrade pulmonary flow, and the source of pre-Glenn pulmonary blood flow. Interestingly, patients with a pre-Glenn pulmonary artery banding showed a smaller amount of SPCF_%PV_ compared to those with a BT-shunt or RV-PA-shunt. Simultaneously, same patients exhibited a significantly higher McGoon ratio compared to those with BT and RV-PA shunts. This suggests that pulmonary artery banding promotes better pulmonary arterial growth, which may decrease the development of SPCF.

In agreement with others (1, 4, 14), SPCF_%PV_ showed a negative association with the pulmonary arterial size in univariate analysis. However, in the multivariate analysis pulmonary arterial size was not associated with SCPF. This may be related to the fact that our study population was a cohort of consecutive single ventricle patients referred for CMR during routine follow-up and the number of patients with pulmonary arterial stenosis was small; only 6/158 (4%) patients had pulmonary arterial stenosis requiring intervention after the CMR examination.

SPCF_%PV_ was not associated with a lower ventricular ejection fraction, nor with the univentricular ventricle volumes, or atrioventricular valve regurgitation. However, some researchers (4, 11, 15) have observed a positive association between SPCF and EDV. In our study, the median follow-up time after TCPC was 11.5 years, and we cannot exclude the possibility that SPCF might have a greater clinical impact on these findings later in adulthood. Further, Kodama et al. (3) revealed a significant positive relationship between the central venous pressure and SPCF%_PV._ However, in our cohort, there was no association between the peripheral venous pressure and SPCF%_PV_.

In recent years, there has been increased interest in understanding the role of neck and thorax lymphatic collaterals in patients with a single ventricle (8, 16, 17). The pathophysiology of the lymphatic system in univentricular patients is not well understood, and the association between SPCF and neck lymphatic collaterals has not been tested widely. In our study, SPCF_%PV_ was not associated with the presence of neck or thorax lymphatic abnormalities, nor with their grade, which agrees with the recent findings of Segar et al. (14).

In our study, we observed that the calculated SPCF ranged from 0.3 to 0.7 L/min/m^2^, with a median of 0.5 L/min/m^2^, which is consistent with previous reports (7, 10, 18, 19). However, we observed a substantial disparity in pulmonary flow when measured from the pulmonary arteries or the pulmonary veins. The largest difference in pulmonary flow in one patient was Q_PA_ 0.5 L/min/m^2^ vs. Q_PV_ 4.0 L/min/m^2^. Such a significant difference in the source of pulmonary flow may introduce error in estimation of Qp by Fick method during catheterization (7, 20). While the Fick method focuses on a single source of pulmonary blood flow, CMR enables the distinct measurement of antegrade pulmonary flow and pulmonary venous flow. Yet, Fang et al. (20) revealed that SPCF exceeding 20% leads to an underestimated antegrade pulmonary flow Qp/Qs ratio during catheterization in the pre-TCPC stage, supporting combining both CMR and catheterization data to accurately estimate PVRi in univentricular patients with high SPCF_%_.

The precise destination within the pulmonary vascular bed to which SPCF is channeled remains shrouded in uncertainty, thus obscuring the understanding of their impact on pulmonary vascular resistance. As of our current understanding, there exists no consensus on whether to utilize CMR measurements derived from the pulmonary artery or pulmonary vein for the calculation of PVRi in hybrid data. Within our cohort, we observed six patients with high SPCF%_PV_ (Table 6). Notably, in three of them, the hybrid PVRi, calculated from pulmonary artery Qp values, ranged from 5.6 to 12 WU.m^2^, while CMR pulmonary veins based or solely Fick method based PVRi calculations ranged from 1.5 to 3.3 WU.m^2^. The clinical scenarios of these three patients lend support to the notion of using antegrade pulmonary flow based Qp measurements in these calculations. Specifically, one of these patients underwent transplantation due to the failing Fontan circulation, another was deemed unsuitable for TCPC for clinically evident pulmonary hypertension, and the third required an urgent operation due to a restrictive atrial septum. Consequently, our findings emphasize the critical need for further investigation into this matter, given the paramount importance of PVRi as an outcome parameter for these patients.

Limitations of the study include the potential for subjective distortion despite the confirmation and validation of Qp and Qs through multiple measurements during the examination by two experienced operators. Additionally, the direct measurement of the volume of small veno-venous collaterals to the pulmonary vein was not possible. However, we believe that any errors in the calculations are likely to be small due to the immediate measurement of peripheral saturation and peripheral venous pressure prior to the CMR examination. For the PVRi calculations, CMR and catheterizations were not simultaneous. However, we contend that the temporal gap between the studies may hold minor clinical significance within this patient group. A strength of this nationwide cohort study was the inclusion of a reasonably sized study population.

In conclusion, SPCF leads to a significant left-to-right shunt, and subsequently decreases spontaneously after the TCPC. We found for pre-TCPC patients, an SPCF%_PV_ threshold of 42% (sensitivity 100%, specificity 80%), and for the post-TCPC group, a threshold of 34% (sensitivity 100%, specificity 98%) in identifying reduced antegrade pulmonary flow. Through the establishment of these SPCF thresholds, our study offers valuable insights into the relationship between SPCF and antegrade pulmonary flow in patients with a single ventricle. The integration of both CMR and cardiac catheterization data could provide a more precise hemodynamic assessment for single ventricle patients with elevated SPCF_%PV_ levels, ultimately resulting in enhanced clinical management and informed decision-making. Our findings highlight the urgent need for further research on accurate hybrid PVRi calculations, as they are crucial for these patients.

## Data Availability

The raw data supporting the conclusions of this article will be made available by the authors, without undue reservation.
