# Supplementary material for: Systemic-to-pulmonary collateral flow associations with antegrade pulmonary flow in single ventricle patients: insights from cardiac magnetic resonance imaging
Source: Front Cardiovasc Med. 2024 Feb 22;11:1304087. doi: 10.3389/fcvm.2024.1304087 (PMC10917948; doi:10.3389/fcvm.2024.1304087)

Supplementary Material

Table S1: Summary of magnetic-resonance sequences parameters*

Sequence	Acquisition voxel size, mm^3	Respiratory compensation	Cardiac gating	Averages	Parallel imaging factor	TR, ms	TE, ms	Pixel bandwidth, Hz/px	Typical acquisition duration
**3D bSSFP**	1.4 x 1.4 x 1.3	Navigator	ECG	1	2 (PE) / 1 (SL) or 3.4 (CS)	4.8–5.0	2.4–2.5	543	4 min 20 s
**bSSFP CINE**	2.0 x 2.1-2.5 x 6.0	Breath-holding	ECG	1	2 (PE) or 2.3 (CS)	3.2	1.6	1,125–1,461	4 s/slice
**2D FLASH with through-plane flow encoding**	1.8 x 2.2-2.6 x 6.0	Free breathing	ECG	3	2 (PE) or 4.0 (CS)	4.6–4.9	2.8–3.2	723–746	30 s/slice
**4D TRAK**	1.7 x 1.7 x 2.0-2.1	Breath-holding	None	1	3 (PE) / 2 (SL) or 5.3 (CS)	3.2–3.8	1.1–1.5	320–1,210	‘7 s/ref.frame 1.7 s/dyn. frame’
‘CS’ = compressed SENSE; ‘PE’ = phase encoding direction; ‘SL’ = slice encoding direction; ‘ECG’ = electrocardiogram; ‘TR’ = repetition time; ‘TE’ = echo time									

* MRI scanner parameters were previously described in Yanovskiy A, Martelius L, Rahkonen O, Pihkala J, Happonen JM, Boldt T, Jaakkola I, Peltonen J, Kortesniemi M, Mattila I, Ojala T. Institutional transition from invasive to non-invasive imaging in children with univentricular heart defects: safety and cost savings. Cardiol Young. 2022 Aug 22:1-7. doi: 10.1017/S1047951122002207. Epub ahead of print. PMID: 35993406.

Table S2. ROC analysis, entire cohort (AUC 0.97), SPCF%$_{PV}$ with sensitivity/specificity ≥80%

SPCF$_{\%PV}$	Sensitivity, %	Specificity, %	Youden index	Nearest upper left corner
**23**	100	80	0,80	0,20
**24**	100	81	0,81	0,19
**24**	100	82	0,82	0,18
**25**	100	82	0,82	0,18
**25**	100	84	0,84	0,16
**25**	100	85	0,85	0,16
**26**	100	85	0,85	0,15
**26**	100	86	0,86	0,14
**27**	100	87	0,87	0,13
**28**	100	87	0,87	0,13
**29**	100	88	0,88	0,12
**30**	100	89	0,89	0,11
**32**	100	89	0,89	0,11
**32**	100	90	0,90	0,10
**34**	**100**	**91**	**0,91**	**0,09**
**36**	94	91	0,85	0,11
**36**	94	92	0,85	0,11
**37**	94	92	0,86	0,10
**38**	81	92	0,74	0,20
**39**	81	93	0,74	0,20

Table S3. ROC analysis, pre-TCPC, (AUC 0.97)

SPCF%PV	Sensitivity,%	Specificity,%	Youden index	Nearest upper left corner
5	100	0	0,00	1,00
9	100	5	0,05	0,95
13	100	10	0,10	0,90
14	100	15	0,15	0,85
15	100	20	0,20	0,80
19	100	25	0,25	0,75
24	100	30	0,30	0,70
26	100	35	0,35	0,65
26	100	40	0,40	0,60
29	100	45	0,45	0,55
34	100	50	0,50	0,50
38	100	55	0,55	0,45
39	100	60	0,60	0,40
40	100	65	0,65	0,35
41	100	70	0,70	0,30
42	100	75	0,75	0,25
**42**	**100**	**80**	**0,80**	**0,20**
43	86	85	0,71	0,21
44	86	90	0,76	0,17
45	86	95	0,81	0,15
47	71	95	0,66	0,29
50	71	100	0,71	0,29
55	57	100	0,57	0,43
58	43	100	0,43	0,57
63	29	100	0,29	0,71
77	14	100	0,14	0,86
89	0	100	0,00	1,00

Table S4. ROC analysis, post-TCPC, (AUC 0.99), SPCF%$_{PV}$ with sensitivity/specificity ≥80%

SPCF%PV	Sensitivity,%	Specificity,%	Youden index	Nearest upper left corner
20	100	80	0,80	0,21
20	100	80	0,80	0,20
20	100	80	0,80	0,20
20	100	83	0,83	0,17
21	100	84	0,84	0,16
21	100	84	0,84	0,16
21	100	85	0,85	0,15
22	100	86	0,86	0,14
23	100	87	0,87	0,13
23	100	89	0,89	0,12
24	100	89	0,89	0,11
24	100	90	0,90	0,10
25	100	91	0,91	0,09
25	100	92	0,92	0,08
25	100	93	0,93	0,07
26	100	93	0,93	0,07
28	100	94	0,94	0,06
29	100	95	0,95	0,05
31	100	96	0,96	0,04
32	100	97	0,97	0,03
**34**	**100**	**98**	**0,98**	**0,03**
36	89	98	0,86	0,11
37	89	98	0,87	0,11
39	67	98	0,65	0,33
41	56	98	0,54	0,44
44	44	98	0,43	0,56
45	33	98	0,32	0,67
48	33	100	0,33	0,67
53	22	100	0,22	0,78
56	11	100	0,11	0,89
58	0	100	0,00	1,00

Figure S1. Scatter plots of hemodynamic and anatomical parameters in the pre-TCPC patients

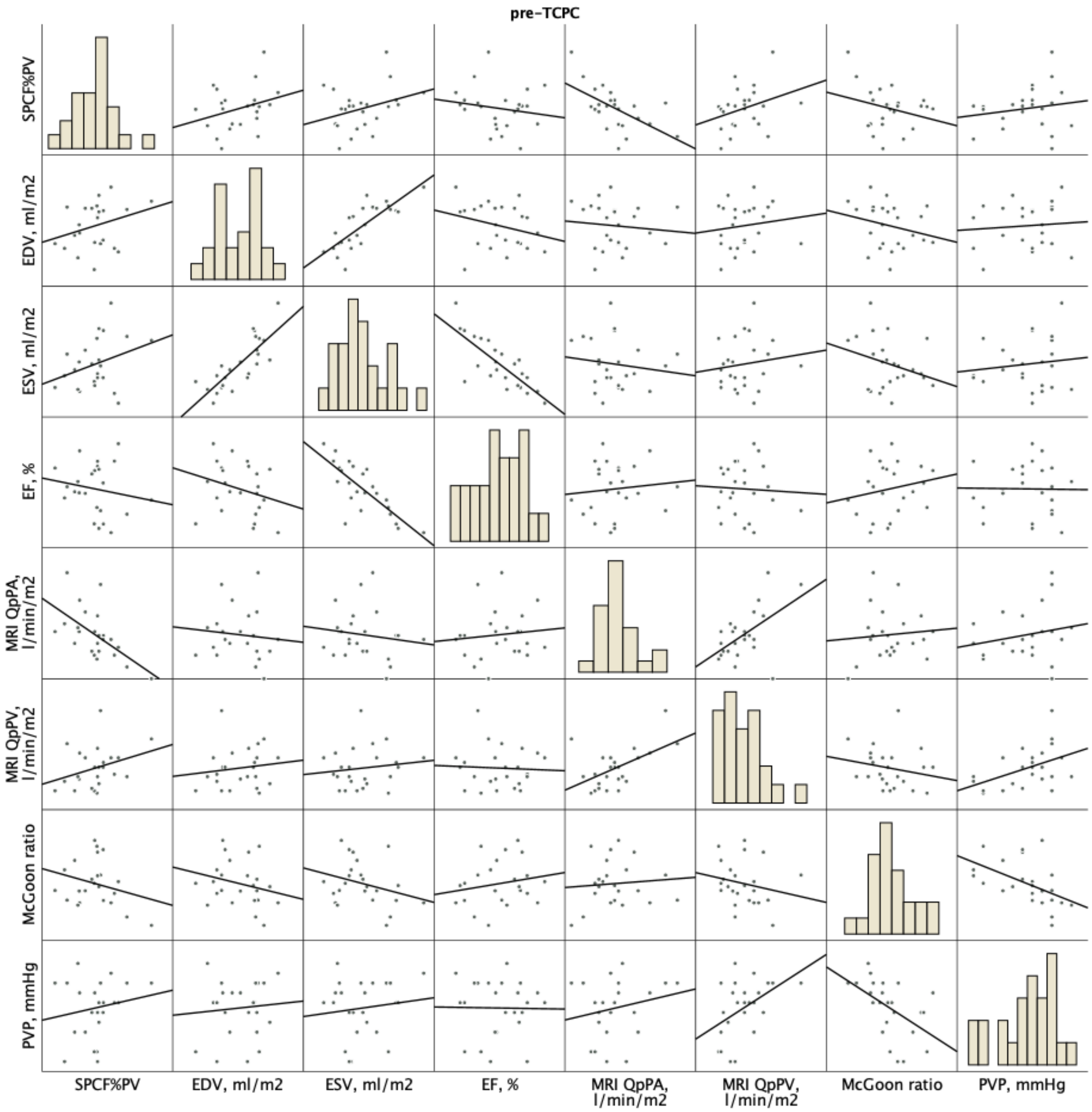

Figure S2. Scatter plots of hemodynamic and anatomical parameters in the post-TCPC patients

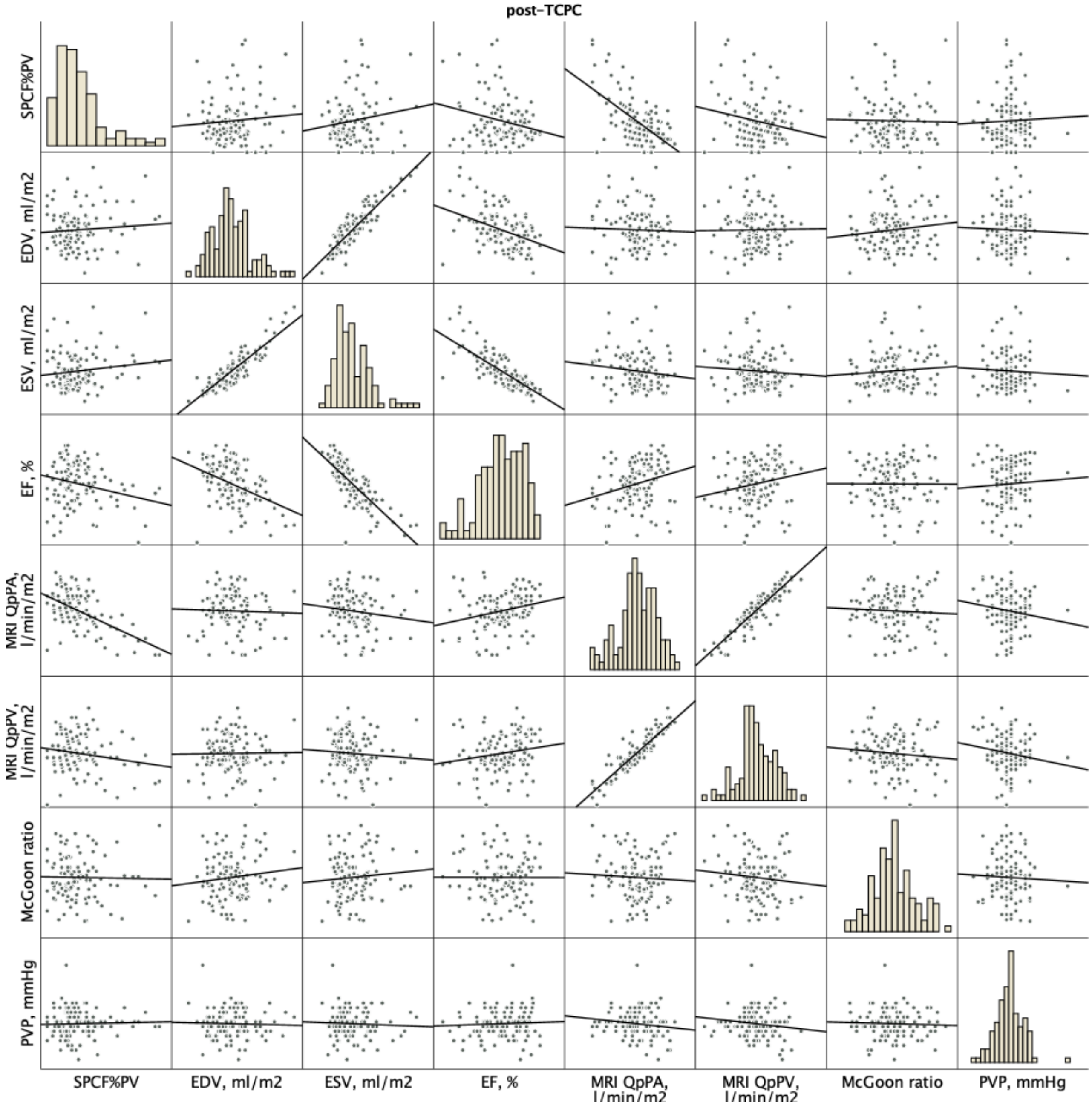

Supplement: Supplementary file 1 [file Datasheet1.pdf]
