# Supplementary material for: Systemic-to-pulmonary collateral flow associations with antegrade pulmonary flow in single ventricle patients: insights from cardiac magnetic resonance imaging
Source: Front Cardiovasc Med. 2024 Feb 22;11:1304087. doi: 10.3389/fcvm.2024.1304087 (PMC10917948; doi:10.3389/fcvm.2024.1304087)

Supplementary Material

**Table S1: Summary of magnetic-resonance sequences parameters***

| **Sequence** | **Acquisition voxel size, mm^3^** | **Respiratory compensation** | **Cardiac gating** | **Averages** | **Parallel imaging factor** | **TR, ms** | **TE, ms** | **Pixel bandwidth, Hz/px** | **Typical acquisition duration** |
| --- | --- | --- | --- | --- | --- | --- | --- | --- | --- |
| **3D bSSFP** | 1.4 x 1.4 x 1.3 | Navigator | ECG | 1 | 2 (PE) / 1 (SL) or 3.4 (CS) | 4.8–5.0 | 2.4–2.5 | 543 | 4 min 20 s |
| **bSSFP CINE** | 2.0 x 2.1-2.5 x 6.0 | Breath-holding | ECG | 1 | 2 (PE) or 2.3 (CS) | 3.2 | 1.6 | 1,125–1,461 | 4 s/slice |
| **2D FLASH with through-plane flow encoding** | 1.8 x 2.2-2.6 x 6.0 | Free breathing | ECG | 3 | 2 (PE) or 4.0 (CS) | 4.6–4.9 | 2.8–3.2 | 723–746 | 30 s/slice |
| **4D TRAK** | 1.7 x 1.7 x 2.0-2.1 | Breath-holding | None | 1 | 3 (PE) / 2 (SL) or 5.3 (CS) | 3.2–3.8 | 1.1–1.5 | 320–1,210 | ‘7 s/ref.frame 1.7 s/dyn. frame’ |
| ‘CS’ = compressed SENSE; ‘PE’ = phase encoding direction; ‘SL’ = slice encoding direction; ‘ECG’ = electrocardiogram; ‘TR’ = repetition time; ‘TE’ = echo time | | | | | | | | | |

* MRI scanner parameters were previously described in Yanovskiy A, Martelius L, Rahkonen O, Pihkala J, Happonen JM, Boldt T, Jaakkola I, Peltonen J, Kortesniemi M, Mattila I, Ojala T. Institutional transition from invasive to non-invasive imaging in children with univentricular heart defects: safety and cost savings. Cardiol Young. 2022 Aug 22:1-7. doi: 10.1017/S1047951122002207. Epub ahead of print. PMID: 35993406.

**Table S2.** ROC analysis, entire cohort (AUC 0.97), SPCF%_PV_ with sensitivity/specificity ≥80%

| SPCF_%PV_ | Sensitivity, % | Specificity, % | Youden index | Nearest upper left corner |
| --- | --- | --- | --- | --- |
| 23 | 100 | 80 | 0,80 | 0,20 |
| 24 | 100 | 81 | 0,81 | 0,19 |
| 24 | 100 | 82 | 0,82 | 0,18 |
| 25 | 100 | 82 | 0,82 | 0,18 |
| 25 | 100 | 84 | 0,84 | 0,16 |
| 25 | 100 | 85 | 0,85 | 0,16 |
| 26 | 100 | 85 | 0,85 | 0,15 |
| 26 | 100 | 86 | 0,86 | 0,14 |
| 27 | 100 | 87 | 0,87 | 0,13 |
| 28 | 100 | 87 | 0,87 | 0,13 |
| 29 | 100 | 88 | 0,88 | 0,12 |
| 30 | 100 | 89 | 0,89 | 0,11 |
| 32 | 100 | 89 | 0,89 | 0,11 |
| 32 | 100 | 90 | 0,90 | 0,10 |
| 34 | **100** | **91** | **0,91** | **0,09** |
| 36 | 94 | 91 | 0,85 | 0,11 |
| 36 | 94 | 92 | 0,85 | 0,11 |
| 37 | 94 | 92 | 0,86 | 0,10 |
| 38 | 81 | 92 | 0,74 | 0,20 |
| 39 | 81 | 93 | 0,74 | 0,20 |

**Table S3.** ROC analysis, pre-TCPC, (AUC 0.97)

| **SPCF%PV** | **Sensitivity,%** | **Specificity,%** | **Youden index** | **Nearest upper left corner** |
| --- | --- | --- | --- | --- |
| 5 | 100 | 0 | 0,00 | 1,00 |
| 9 | 100 | 5 | 0,05 | 0,95 |
| 13 | 100 | 10 | 0,10 | 0,90 |
| 14 | 100 | 15 | 0,15 | 0,85 |
| 15 | 100 | 20 | 0,20 | 0,80 |
| 19 | 100 | 25 | 0,25 | 0,75 |
| 24 | 100 | 30 | 0,30 | 0,70 |
| 26 | 100 | 35 | 0,35 | 0,65 |
| 26 | 100 | 40 | 0,40 | 0,60 |
| 29 | 100 | 45 | 0,45 | 0,55 |
| 34 | 100 | 50 | 0,50 | 0,50 |
| 38 | 100 | 55 | 0,55 | 0,45 |
| 39 | 100 | 60 | 0,60 | 0,40 |
| 40 | 100 | 65 | 0,65 | 0,35 |
| 41 | 100 | 70 | 0,70 | 0,30 |
| 42 | 100 | 75 | 0,75 | 0,25 |
| **42** | **100** | **80** | **0,80** | **0,20** |
| 43 | 86 | 85 | 0,71 | 0,21 |
| 44 | 86 | 90 | 0,76 | 0,17 |
| 45 | 86 | 95 | 0,81 | 0,15 |
| 47 | 71 | 95 | 0,66 | 0,29 |
| 50 | 71 | 100 | 0,71 | 0,29 |
| 55 | 57 | 100 | 0,57 | 0,43 |
| 58 | 43 | 100 | 0,43 | 0,57 |
| 63 | 29 | 100 | 0,29 | 0,71 |
| 77 | 14 | 100 | 0,14 | 0,86 |
| 89 | 0 | 100 | 0,00 | 1,00 |

**Table S4.** ROC analysis, post-TCPC, (AUC 0.99), SPCF%_PV_ with sensitivity/specificity ≥80%

| **SPCF%PV** | **Sensitivity,%** | **Specificity,%** | **Youden index** | **Nearest upper left corner** |
| --- | --- | --- | --- | --- |
| 20 | 100 | 80 | 0,80 | 0,21 |
| 20 | 100 | 80 | 0,80 | 0,20 |
| 20 | 100 | 80 | 0,80 | 0,20 |
| 20 | 100 | 83 | 0,83 | 0,17 |
| 21 | 100 | 84 | 0,84 | 0,16 |
| 21 | 100 | 84 | 0,84 | 0,16 |
| 21 | 100 | 85 | 0,85 | 0,15 |
| 22 | 100 | 86 | 0,86 | 0,14 |
| 23 | 100 | 87 | 0,87 | 0,13 |
| 23 | 100 | 89 | 0,89 | 0,12 |
| 24 | 100 | 89 | 0,89 | 0,11 |
| 24 | 100 | 90 | 0,90 | 0,10 |
| 25 | 100 | 91 | 0,91 | 0,09 |
| 25 | 100 | 92 | 0,92 | 0,08 |
| 25 | 100 | 93 | 0,93 | 0,07 |
| 26 | 100 | 93 | 0,93 | 0,07 |
| 28 | 100 | 94 | 0,94 | 0,06 |
| 29 | 100 | 95 | 0,95 | 0,05 |
| 31 | 100 | 96 | 0,96 | 0,04 |
| 32 | 100 | 97 | 0,97 | 0,03 |
| **34** | **100** | **98** | **0,98** | **0,03** |
| 36 | 89 | 98 | 0,86 | 0,11 |
| 37 | 89 | 98 | 0,87 | 0,11 |
| 39 | 67 | 98 | 0,65 | 0,33 |
| 41 | 56 | 98 | 0,54 | 0,44 |
| 44 | 44 | 98 | 0,43 | 0,56 |
| 45 | 33 | 98 | 0,32 | 0,67 |
| 48 | 33 | 100 | 0,33 | 0,67 |
| 53 | 22 | 100 | 0,22 | 0,78 |
| 56 | 11 | 100 | 0,11 | 0,89 |
| 58 | 0 | 100 | 0,00 | 1,00 |

**Figure S1.** Scatter plots of hemodynamic and anatomical parameters in the pre-TCPC patients


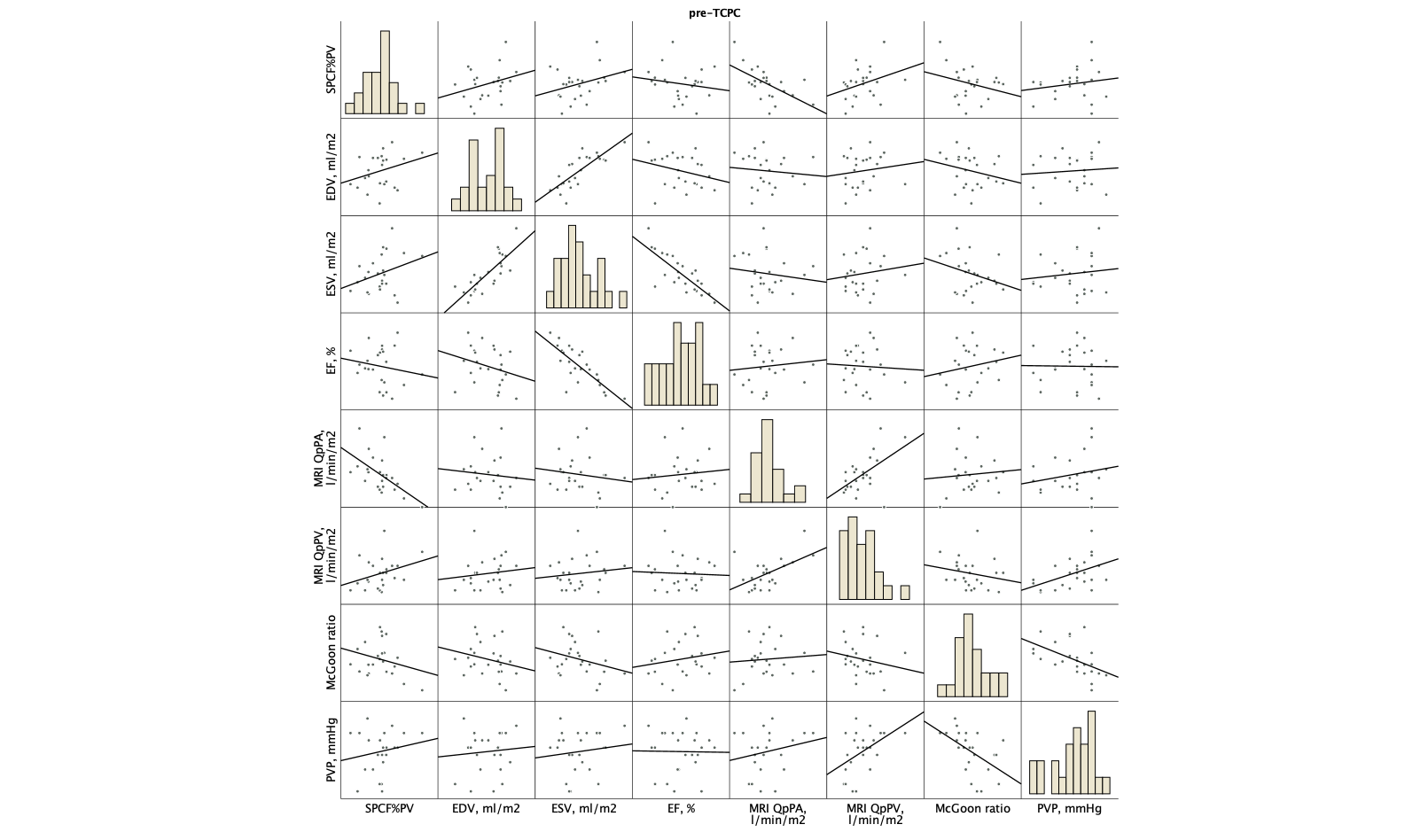


**Figure S2.** Scatter plots of hemodynamic and anatomical parameters in the post-TCPC patients


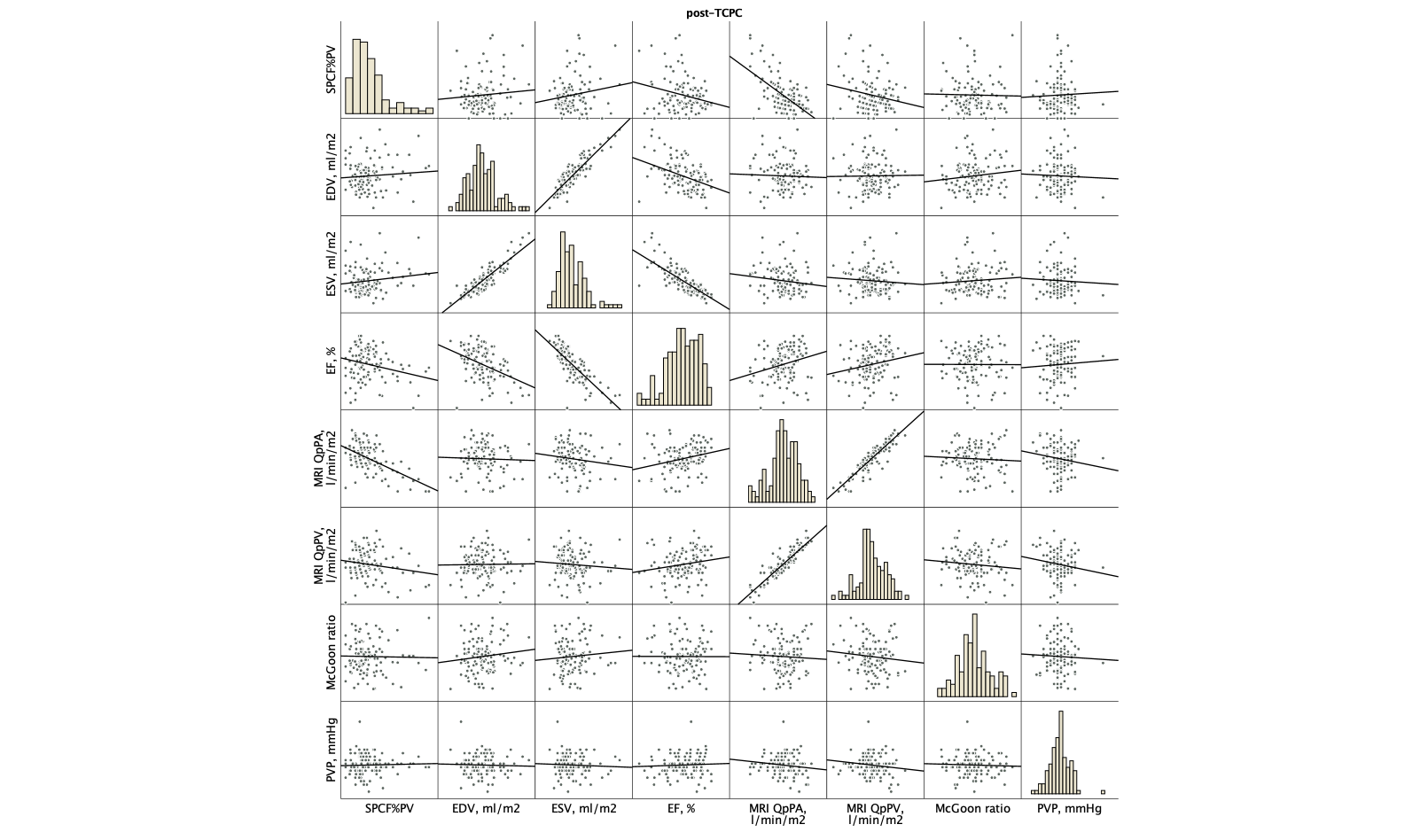

Supplement: Supplementary file 2 [file Table1.docx]
